# Diffuse bullous cutaneus mastocytosis in an indigenous child of the Amazon Region of Ecuador

**DOI:** 10.1186/1939-4551-8-S1-A250

**Published:** 2015-04-08

**Authors:** Gabriela Varsovia Acosta Ruiz, Pedro Alfonso Cueva Estrada, Margarita Guadalupe Acosta Ruiz

**Affiliations:** 1 European Academy of Allergy and Clinicall Immunology, Spain; 2 Sociedad Ecuatoriana De Alergología e Inmunología Clínica, Ecuador; 3 Hospital General Puyo, Ecuador; 4 Asociación Ecuatoriana De Dermatología y Ciencias Afines, Ecuador; 5 Colegio Médico De Pichincha, Ecuador

## Background

Diffuse Bullous Mastocytosis is a very rare variant of Cutaneous Mastocytosis. It is characterized by a diffuse infiltration of the skin by mast cells manifesting as yellowish, thickened doughy skin with appearance of large blisters. This is the first report of a case of this condition in an indigenous child of The Amazon region of Ecuador.

## Methods

We report a 9-year-old female infant with history of recurrent episodes of vesicles bullous lesions on bilateral upper and lower extremities since 4 months old. She has presented four widespread critical episodes that required treatment in an Emergency department over the last 9 years. She had only received primary care attention.

When the patient consulted at the Dermatology outpatient department, She presented generalized dermatosis characterized by a polymorphism injury: urticarial wheals, erythematous vesicles and blisters, postinflammatory hyperpigmented macules, facial and eyelid angioedema, moderate pruritus. Also, target lesions on palms and soles.

Systemic examination was within normal limits.

Skin biopsy from an axilar lesion showed subepidermal bulla and an upper dermal inflammatory infiltrate comprising of lymphocytes and many mast cells. The overlying epidermis with spongiosis and intraepidermal blister. It was diagnosed as Difusse Cutaneus Mastocytosis and the child was remited to the Allergy departament.

## Results

After 3 weeks of short course of prednisolone (1mg/kg) along with H1 and H2 receptor antagonists, the control of the symptoms was obtained. General and specific recommendations were also provided.

## Conclusions

This is the first report of a case of Diffuse Bullous Mastocytosis in an indigenous child of The Amazon region of Ecuador. This diagnosis was made possible by the improvement in health services atention specialist trained in the Amazon region of Ecuador.

